# Brain Activity Stimulated by Prism Adaptation Tasks Utilized for the Treatment of Unilateral Spatial Neglect: A Study with fNIRS

**DOI:** 10.1155/2012/312781

**Published:** 2012-04-23

**Authors:** Hiroshi Taniguchi, Makoto Hiyamizu, Takanori Tominaga, Shu Morioka

**Affiliations:** ^1^Department of Neurorehabilitation, Graduate School of Health Science, Kio University, 4-2-2 Umami-naka koryo-cho, Kitakatsuragi-gun, Nara 635-0832, Japan; ^2^Department of Rehabilitation, Murata Hospital, 4-2-1 Tashima-cho Ikuno-ku Osaka 544-0011, Japan

## Abstract

We investigated the neurological basis for efficacy of prism adaptation therapy, which is used for the treatment of poststroke unilateral spatial neglect (USN). Study subjects were 6 USN-positive (+), 6 USN-negative patients, and 6 healthy volunteer control subjects. USN was identified by the Behavioural Inattention Test (BIT). During the tasks, brain activity was assessed with fNIRS via changes in oxyHb concentration per unit length. There was no significant difference in the number of errors in the task between the 3 groups. However, in the USN(+) group there was a significantly greater reduction in oxyHb levels in the right parietal association cortex during the prism adaptation task than in the other 2 groups (*P* < 0.05). There was an immediate improvement in USN symptoms as well as a significant increase in oxyHb levels during the prism adaptation in the channels covering the right frontal and parietal lobes in 2 patients in the USN(+) group (*P* < 0.05). This result suggested that decreased activity in the right parietal association cortex, which is related to spatial perception, during the prism adaptation task and task-induced reorganization of the right frontal and parietal areas were involved in improvement in USN symptoms.

## 1. Introduction

Recently, it was shown that prism adaptation therapy not only instantly improved unilateral spatial neglect (USN) symptoms but also enhanced balancing ability in patients with USN after stroke [1, 2]. Also reported has been the use of prism adaptation therapy for the treatment of USN to achieve improvement in USN symptoms as well as in movements [1–3]. This therapy is aimed at inducing the USN patient to pay attention to the neglect space by reaching out with an upper limb toward an object using deviated visual information caused by a prism [1].

 The prism deviates the visual information so soon after the start of the task that the patient cannot precisely reach an object. However, repeated attempts enable the patient to successfully reach the object through adaptive learning [4]. When the patient attempts such reaching movements under normal visual conditions without prism-mounted eyeglasses after prism adaptation therapy, the patient's motion deviates to the opposite side of the object as an aftereffect. Hence, the prism adaptation task has a physical exercise component as well as an adaptive process of feedback control of deviated movements so that the brain is forced to correct or reorganize spatial perception.

 Neural mechanisms of action for these prism adaptation effects have been reported exclusively in healthy subjects by studies using brain imaging modalities as mentioned below but very few reported in USN cases. According to these studies, the posterior parietal lobe was observed by one group to be selectively activated at the time of reaching movements, while the subject was wearing prism-mounted eyeglasses [5]. On the other hand, it was found that the right premotor area and cerebellum were activated when adaptive learning slowly progressed besides doing reaching movements [6]. It was clarified using functional magnetic resonance imaging (fMRI) that brain activation took place only in the anterior area of the interparietal sulcus at the initial phase of adaptation, but that activation was shifted to the posterior area of the sulcus and the cerebellum [7]. These studies in healthy subjects suggest that several brain areas such as the parietal lobe, thalamus, premotor cortex, and cerebellum are involved in prism adaptation effects. In contrast, patients with cerebellar injury were reported to have depressed prism adaptation, namely, to have problems with learning related to movement [8].

From these studies, it is inferred that prism adaptation therapy produces its effect through activation of brain areas such as the cerebellum, parietal lobe, premotor cortex, and thalamus. On the other hand, it remains unclear how the brain is activated as USN symptoms are improved by prism adaptation therapy, which has been studied in terms of improvement in symptoms. Previous brain imaging studies were performed primarily with healthy subjects but not with poststroke USN cases. Moreover, a comparison between USN-positive (+) and USN-negative (−) stroke patients has not been made. The present study, therefore, aimed to clarify through the use of functional near-infrared spectroscopy (fNIRS), which enables measurement of brain activity in practically free daily life, how brain activity differs between USN(+) and USN(−) stroke patients at the time of prism adaptation, and how brain activity changes during the process of immediate improvement in USN symptoms.

## 2. Methods

### 2.1. Study Subjects

Subjects were 12 patients with poststroke injury of the right hemisphere who were administered the Japanese version of the Behavioural Inattention Test (BIT) for detection of USN. One-half was found to have USN (6 patients, mean age  69 ± 6.6 y, disease duration 17.0 ± 19.6 mo), while the other half was found to be free from USN (6 patients, mean age 59.8 ± 11.2 y, disease duration 18.8 ± 14.0 mo) (Table 1). Cutoff values for the BIT were set at 131 points for the ordinary test (the dichotomy of the behavioural subtests of the BIT) and at 68 for the action test [9], with those scoring below the cutoff value determined to have USN. All subjects had normal intelligence. Six healthy subjects who were free of age-associated decline in visual acuity and who had normal intelligence (mean age 24.5 ± 1.6) comprised the control group. I compared it in these three groups.

We described the experimental protocol of the present study to all participants after which they provided informed consent to take part in the study.

### 2.2. Procedures for the Prism Adaptation Task

The prism adaptation task involved reaching with an upper limb toward a visible object while seated on a chair. The subject was requested to extend the right upper limb toward a target (rod) at 45 cm in front of the subject starting from a point at 5 cm in front of the subject. Every subject attempted the task under dual conditions: wearing or not wearing prism-mounted eyeglasses (adaptation or control, resp.). The prism-mounted eyeglasses deviated the subject's line of sight by 10° toward the right side, necessitating the subject to adjust the visual information in the process. The subject was prevented from visually confirming the actual path of the reaching movement before the task was imposed. Three sets of the task were imposed, with each set consisting of a 10 s rest period, 20 s of reaching motion, and another 10 s rest period. Reaching movements were controlled using a metronome, with one motion attempt per second, with a total of 20 actions per set.

 Failed attempts at successfully reaching the object were counted as errors. The USN(+) group was examined with the BIT before and after the task to evaluate improvement in USN.

### 2.3. NIRS Measurements

**Figure 1:**
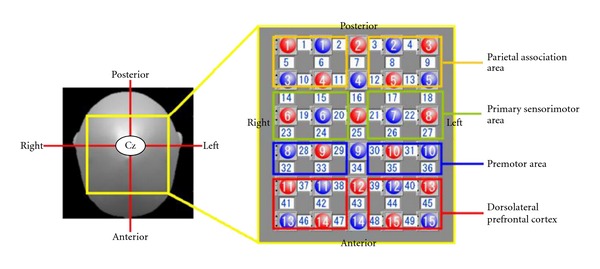
Measurement by functional near infrared spectroscopy. We employed a 49-channel system with 30 optodes. 15 light sources (red numbers) and 15 detectors (blue numbers) that covered the frontoparietal area. Solid white numbers denote measuring channels, which were spread to 8 regions of interest.

The fNIRS system (FOIRE-3000; Shimadzu, Kyoto, Japan) with continuous wave laser diodes with wavelengths of 780, 805 and 830 nm was used to record cortical activity at a sampling rate of 5 Hz. In brief, we employed a 49-channel system with 30 optodes (15 light sources and 15 detectors). This system detected changes in the cortical concentration levels (mM cm) of oxygenated hemoglobin (oxyHb), deoxygenated hemoglobin (deoxyHb), and total hemoglobin by applying the modiWed Beer-Lambert law. The optodes were positioned using the International 10/20 system with Cz located beneath the 7th light source and the other ones located at intervals of 3.0 cm centering on this 7th light source. The fNIRS topographic map covered the frontoparietal area, which was divided into 8 regions of interest (Figure 1) based on the functional anatomy of the parietal and prefrontal regions. The left sensorimotor cortex (SMC) was covered by channels 17, 18, 21, 22, 26, and 27; the right SMC by channels 14, 15, 19, 20, 23, and 24; and the left and right pre motor cortex (PMC) by channels 30, 31, 35, and 36 and channels 28, 29, 32, and 33, respectively; the left and right PFC by channels 37, 38, 41, 42, 46, and 47 and channels 39, 40, 44, 45, 48, and 49, respectively; and the left and right parietal area by channels 3, 4, 8, 9, 12, and 13 and 1, 2, 5, 6, 10, and 1, respectively (Figure 1).

### 2.4. NIRS Data Analyses

We selected oxyHb levels as markers of cortical activity because oxyHb is the most sensitive indicator of locomotion-related changes in regional cerebral blood flow [10–12]. Moreover, there are considerable individual differences in task-related changes in deoxyHb levels, probably due to variable neurovascular coupling in the elderly [12–15]. Changes in the oxyHb levels were calculated during the task phases of control and prism adaptation tasks, which were defined as follows: rest phase, 10 s before the beginning of the task period followed by a 20 s task phase. Regional changes in the oxyHb level during the control and prism adaptation phases were obtained from each channel in each subject. Data for 3 repetitions were averaged in each channel; then the value for each region of interest was obtained by averaging data from the several channels (see Figure 1) in each subject. 

To evaluate the effect of cortical activation during the control and prism adaptation periods, we calculated the effect size (ES) to adjust the influence of differential path length factors among subjects and cortical regions on oxyHb levels [16]. The ES for the effect of the prism adaptation task on activities was calculated by the following formula: mean oxyHb value during prism adaptation task—mean oxyHb value during control task/standard deviation of oxyHb value during control task.

## 3. Statistical Analyses

We used software, Dr. SPSS II for Windows, for statistical analyses. Intergroup comparisons of errors made in the prism adaptation task were conducted by one-way ANOVA. Regarding Intergroup comparisons of ES values for each cortical area, one-way ANOVA was employed, and post hoc analysis was made by Bonferroni test. A significance level was set at less than 5%.

In the USN(+) group, patients were categorized into a USN-improved or a USN-unimproved subgroup based on results of the BIT performed after the prism adaptation task and Δ[oxyHb] was calculated by subtracting the oxyHb value under the prism adaptation condition from that under the control condition. Interpatient comparison in fNIRS was made using a significance level calculated by Z-score according to a previous study [17]. Moreover, the significance level (Z-score of >3.28) was calculated from 10 s NIRS data under the control condition after Bonferroni adjustment. A channel that exhibited a significant Δ[oxyHb] level was assumed to reflect a significant change [17]. A response in each channel was deemed significant when the mean oxyHb value during prism adaptation was above the value for the level of significance.

## 4. Results

### 4.1. Prism Adaptation Error

**Figure 2:**
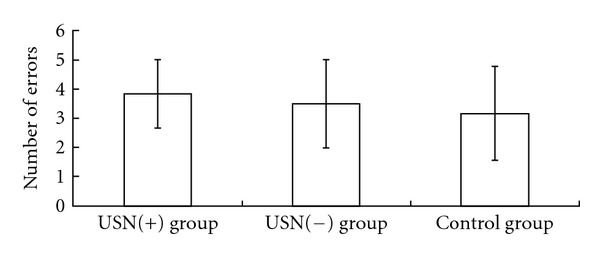
Comparison of the number of errors in the prism adaptation task. There was no significant difference between the 3 groups (*P* = 0.72). USN: unilateral spatial neglect.

In all 3 groups, all subjects successfully reached the target in the prism adaptation task. There was no significant difference between the 3 groups in the number of errors (Figure 2).

### 4.2. Comparison between Groups (ES on fNIRS Measurement)

The parietal association area showed a significant decrease in ES levels in the prism adaptation task in the USN(+) group compared to the control and USN(−) groups (*P* < 0.05, Table 2). There was no significant Intergroup differences in brain activity in other areas.

### 4.3. Comparison between Individuals with USN (BIT Score and Z-Score on fNIRS Measurement)

**Figure 3:**
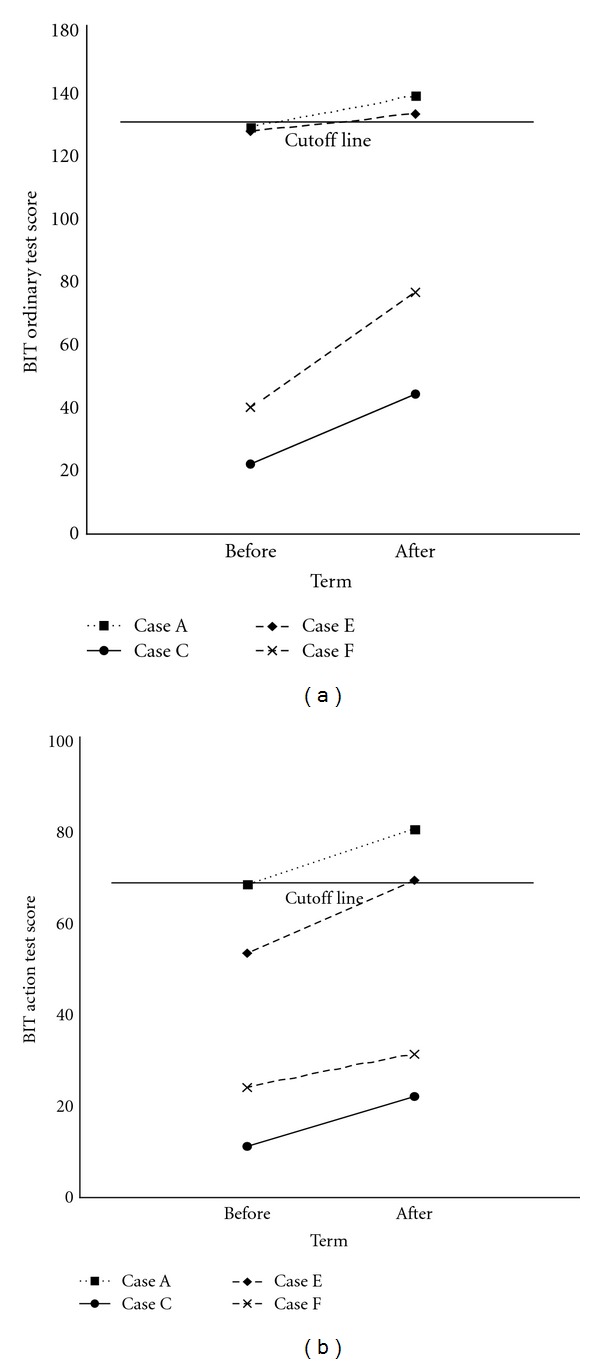
Changes in Behavioural Inattention Test (BIT) scores after the prism adaptation task in the USN(+) group. Cases A and B had higher BIT scores that were over the cutoff value (131 points for the ordinary test and 68 points for the action test) after prism adaptation. (128 to 138 points and 68 to 80 points for the ordinary and action tests, resp., in case A; 127 to 132 and 53 to 69 correspondingly in case B). USN: unilateral spatial neglect.

In the USN(+) group, in 4 of the 6 subjects, BIT points were increased after the task (Figure 3). In 2 subjects (Cases A and E), USN symptoms were improved as evidenced by BIT scores that exceeded the cutoff. Case A scored 138 points in the ordinary BIT and 80 in the action BIT, while Case E scored 132 and 69 in the ordinary and action BIT, respectively. In these 2 cases, the oxyHb levels in the channels covering the right parietal association cortex as well as the dorsolateral region of the right prefrontal cortex were significantly increased during the prism adaptation task compared to the control task. In both of these subjects, there was a significant increase in oxyHb levels in channels 1, 36, and 47 (*P* < 0.05, Table 3). In contrast, there was no significant difference in oxyHb levels in any channel between the control and prism adaptation tasks in the remaining 4 USN(+) subjects.

## 5. Discussion

In this study, there was no significant difference in prism adaptation errors among the control, USN(+) and (−) groups. It was, therefore, considered that prism adaptation effects were observed in all of the subjects, although brain activity during adaptation differed among the groups. Concerning brain activity at the time the prism adaptation task was begun, oxyHb levels decreased in the right parietal association area exclusively in USN(+). It has been reported that the temporoparietal junction [18, 19], frontal lobe [20, 21], putamen, and disorders of the neural network between pulvinar and superior temporal gyrus [22, 23] are implicated in USN development. Also, the right parietal lobe is regarded as an important responsible region. The parietal lobe relays input and output signals of motivation, active exploration, and sensation such as arousal, and Mesulam implicated such neural network disorders in the development of USN [24].

 The right parietal lobe was reported to be activated in healthy persons during the process of recognizing prism adaptation-caused errors in visual as well as somatic sensation and the process of correcting these errors [5, 25]. Consequently, in our USN(+) patients, the right parietal lobe might not be activated sufficiently due to its injury. However, we found interesting correlations between improvement in symptoms and brain activity in the USN(+) cases. Two of the 6 USN(+) cases scored BIT points above the cutoff value after the prism adaptation task, reflecting an instant improvement in USN symptoms. Only these 2 patients had an increase in oxyHb concentration per unit length in the right frontal and parietal lobe areas, whereas such an elevation of the oxyHb concentration per unit length was observed nowhere in brain areas in those who had greater improvements in USN symptoms after the task. It was reported that disorders of the superior longitudinal fasciculus, which connects the frontal lobe to the parietal lobe, are related to USN severity [26]. Accordingly, these 2 patients were considered to have improved USN through enhanced activation of the frontoparietal areas after the prism adaptation task.

 The development of USN is thus related to lowered activity of the right parietal association area. It was suggested that not only activation of the right parietal lobe but also such activation-induced reorganization of its neural networks might work in the improvement of USN [26]. In addition, there is the possibility that prism adaptation might contribute to formation of neural networks. In fact, no activation in these areas was observed in the subjects who failed to show an improvement in USN symptoms as assessed by BIT. According to Corbetta et al., the recovery process in USN requires reorganization of functionally related neural mechanisms as a whole but is not limited to plasticity of localized lesions [27]. We consider that this theory was verified in part by our results that showed immediate USN recovery as well as our observations on brain activity in the present study. A recent randomized controlled trial provided support for the efficacy of prism adaptation therapy and indicated that its effects differed in different grades of USN severity as determined by BIT [28]. This difference may be partly attributed to activation of the right frontoparietal areas at the time of the task.

 Limitations of the present study are the small sample size, failure to continue to followup therapeutic effects and brain activity, and the inability to acquire information on brain activity in regions other than cortical areas due to the restricted feasibility of fNIRS. It is worthy to mention that there was no significant difference in the number of errors between the USN(+) and USN(−) groups. This may be explained by the possibility that error correction was made by the cerebellum, the function of which could not be detected in the present study. This possibility requires further study using an imaging device that can detect cerebellar activity.

## Figures and Tables

**Table 1:** Characteristic of subjects.

Group	Case	Gender	Age	Disease duration (mo)	Scores for ordinary BIT	Scores for action BIT	Lesion
USN(+)	A	Male	65	4	128	68	Parietal lobe
	B	Male	81	31	66	49	Parietolateral lobe
	C	Male	65	5	22	11	Putamen
	D	Male	68	51	105	47	Putamen
	E	Female	72	6	127	53	Putamen
	F	Male	63	5	40	24	Putamen

USN(−)	G	Female	68	32	138	76	Putamen
	H	Female	41	6	144	77	Putamen
	I	Female	63	8	135	73	Putamen
	J	Male	55	36	144	77	Putamen
	K	Male	75	26	143	77	Putamen
	L	Male	55	5	145	81	Occipitotemporal lobe

An ordinary BIT score of 131 or greater together with an action BIT score of 68 or greater determined the presence of USN. There was no significant difference in age (*P* = 0.11) or disease duration (*P* = 0.41) between the USN-positive and -negative groups.

Abbreviations: BIT: Behavioural Inattention Test; USN: unilateral spatial neglect.

**Table 2:** Comparison of effect size between regions of interest (ROIs) in 3 study groups.

ROI	USN(+) group (*n* = 6)	USN(−) group (*n* = 6)	Control group (*n* = 6)	*P* value
Right parietal association area	−0.035 ± 0.203	0.986 ± 0.969	1.113 ± 1.039	0.05^†^
Right primary sensorimotor area	−0.132 ± 0.167	0.196 ± 0.446	0.547 ± 1.086	0.24
Right premotor area	0.024 ± 0.107	0.096 ± 0.243	0.175 ± 0.312	0.68
Right dorsolateral prefrontal cortex	0.048 ± 0.113	−0.273 ± 0.408	2.204 ± 3.612	0.39
Right parietal association area	0.122 ± 0.509	0.500 ± 0.956	0.553 ± 0.628	0.54
Left sensorimotor area	−0.022 ± 0.069	−0.143 ± 1.355	0.221 ± 0.462	0.35
Left premotor area	0.285 ± 0.841	0.334 ± 0.790	0.338 ± 1.787	0.99
Left dorsolateral prefrontal cortex	0.069 ± 0.128	0.290 ± 1.087	−0.306 ± 0.833	0.49

Values are mean ± standard deviation.

^†^: Significantly lower compared to the USN(−) and control groups.

USN: unilateral spatial neglect.

**Table 3:** Changes in oxyHb concentration in the channels in which both cases A and E had a significant difference in concentration between the control and prism adaptation tasks.

	Channel 1		Channel 38		Channel 47	
	Control task	PA task	Control task	PA task	Control task	PA task
Case A	0.654 ± 0.002	0.801 ± 0.001	0.325 ± 0.003	0.433 ± 0.001	0.434 ± 0.004	0.514 ± 0.002
Case E	0.478 ± 0.001	0.492 ± 0.001	0.272 ± 0.002	0.283 ± 0.008	0.331 ± 0.001	0.353 ± 0.002

Values are mean ± standard deviation (mM·mm).

PA task: Prism adaptation task.

Both cases A and E had significantly higher concentrations of oxyHB in channels 1, 38, and 47 during the prism adaptation task than during control task (*P* < 0.05).
